# No evidence for fixation of mesh in laparoscopic transabdominal preperitoneal (TAPP) inguinal hernia repair: a systematic review and meta-analysis of randomized controlled trials

**DOI:** 10.1007/s00464-023-10237-0

**Published:** 2023-09-06

**Authors:** K. A. Riemenschneider, H. Lund, H. C. Pommergaard

**Affiliations:** 1 Department of Surgery and Transplantation, Rigshospitalet, University of Copenhagen, Inge Lehmanns Vej 7, 2100 Copenhagen, Denmark; 2 Department of Surgery, Nordsjaellands Hospital, Dyrehavevej 29, 3400 Hilleroed, Denmark

**Keywords:** Inguinal hernia, Mesh, Fixation, TAPP, Laparoscopic

## Abstract

**Objective:**

To investigate the differences in hernia recurrence and chronic postoperative inguinal pain (CPIP) in randomized, controlled trials comparing fixation and non-fixation of the mesh in laparoscopic transabdominal preperitoneal (TAPP) inguinal hernia repair.

**Methods:**

A multi-database systematic search was conducted for randomized, controlled trials comparing fixation versus non-fixation of the mesh in TAPP inguinal hernia repair. All eligible papers were assessed for risk of bias using the revised Cochrane risk of bias tool for randomized trials (RoB 2.0). Quality of evidence was evaluated using the GRADE system. Meta-analyses were performed regarding recurrence and CPIP using RevMan.

**Results:**

Seven prospective, randomized controlled trials were included. Laparoscopic TAPP inguinal hernia repair was performed in 1732 patients with 737 procedures performed without fixation and 995 procedures with fixation of the mesh. Despite all trials being RCTs, the trials were limited by substantial bias and the quality of evidence was low regarding hernia recurrence and very low regarding CPIP. Pooled estimates from meta-analyses were an OR of 2.80 (95% CI 0.61–12.77) for hernia recurrence and a mean difference in visual analogue scale (VAS) of 0.17 (95% CI 0.90–1.24) for CPIP, respectively.

**Conclusion:**

The current evidence is very uncertain and mesh fixation may have little to no effect regarding hernia recurrence and chronic postoperative inguinal pain in patients operated with TAPP inguinal hernia repair.

**Supplementary Information:**

The online version contains supplementary material available at 10.1007/s00464-023-10237-0.

Among patients undergoing laparoscopic repair of an inguinal hernia, up to 20% have chronic postoperative inguinal pain (CPIP) [1] with hernia recurrence and open inguinal repair being the main risk factors. Therefore, a laparoscopic approach with either laparoscopic total extraperitoneal (TEP) or transabdominal preperitoneal (TAPP) repair is recommended, provided that sufficient laparoscopic expertise is available [2]. 

In a laparoscopic inguinal hernia repair, the mesh can be either fixated or non-fixated. There are various methods of fixation including the use of glue, tackers, sutures or staples. Some types of meshes are self-adhesive. There is no consensus whether to fixate a mesh or not [2]. In a review published in 2012 evaluating TEP/TAPP [3], there was no benefit of fixating the mesh regarding recurrence. The review did not address CPIP. The latest guidelines from the European Hernia Society from 2018 [2] state that non-fixation was recommended, except for large medial hernias.

The aim of the present review was to investigate differences in hernia recurrence and CPIP in randomized, controlled trials comparing fixation and non-fixation of the mesh in TAPP inguinal hernia repair.

## Methods

The current review was reported according to the PRISMA (Preferred Reporting Items of Systematic reviews and Meta-Analyses) statement [4]. Analytical methods and inclusion criteria were specified in advance and registered at PROSPERO (registration no. CRD42020135436). A dedicated protocol prior to initiating the search review process was not completed. However, methods including eligibility criteria and PICOS were decided in the author group prior to performing search and selection studies.

### PICOS

(P)opulation: Patients with inguinal hernias, undergoing surgery with a laparoscopic approach.

(I)ntervention: Laparoscopic transabdominal preperitoneal (TAPP) inguinal hernia repair with fixation.

(C)omparison: Laparoscopic transabdominal preperitoneal (TAPP) inguinal hernia repair without fixation.

(O)utcome: Hernie recurrence and chronic postoperative inguinal pain (CPIP).

(S)tudies: Randomized controlled clinical trials.

### Eligibility criteria

Included studies were required to be prospective, randomized clinical trials written in English, that evaluated hernia recurrence and CPIP in patients following laparoscopic hernia repair, TAPP, comparing fixation and non-fixation of the mesh. Studies on non-inguinal groin hernias were excluded.

The investigated outcomes were hernia recurrence and CPIP. The definition of hernia recurrence was clinical symptoms suggesting hernia recurrence, followed by a clinical examination confirming the diagnosis. CPIP was defined as patients with at least 3 months of postoperative pain located to the groin area. CPIP had to be evaluated using the visual analogue scale (VAS) [5] or one dimensional numerical rating scale (NRS) [6] graded from 0 to 10, to be included in the meta-analysis [7, 8].

PubMed [National Library of Medicine (1966–present)] and EMBASE (1974–present) databases were searched for articles meeting the above-mentioned criteria, on the 24th of January 2021. The development of the search strategy was done as a collaboration between authors KAR and HL. The search strings used in PubMed and EMBASE are provided in Online Appendix 1.

**Fig. 1:**
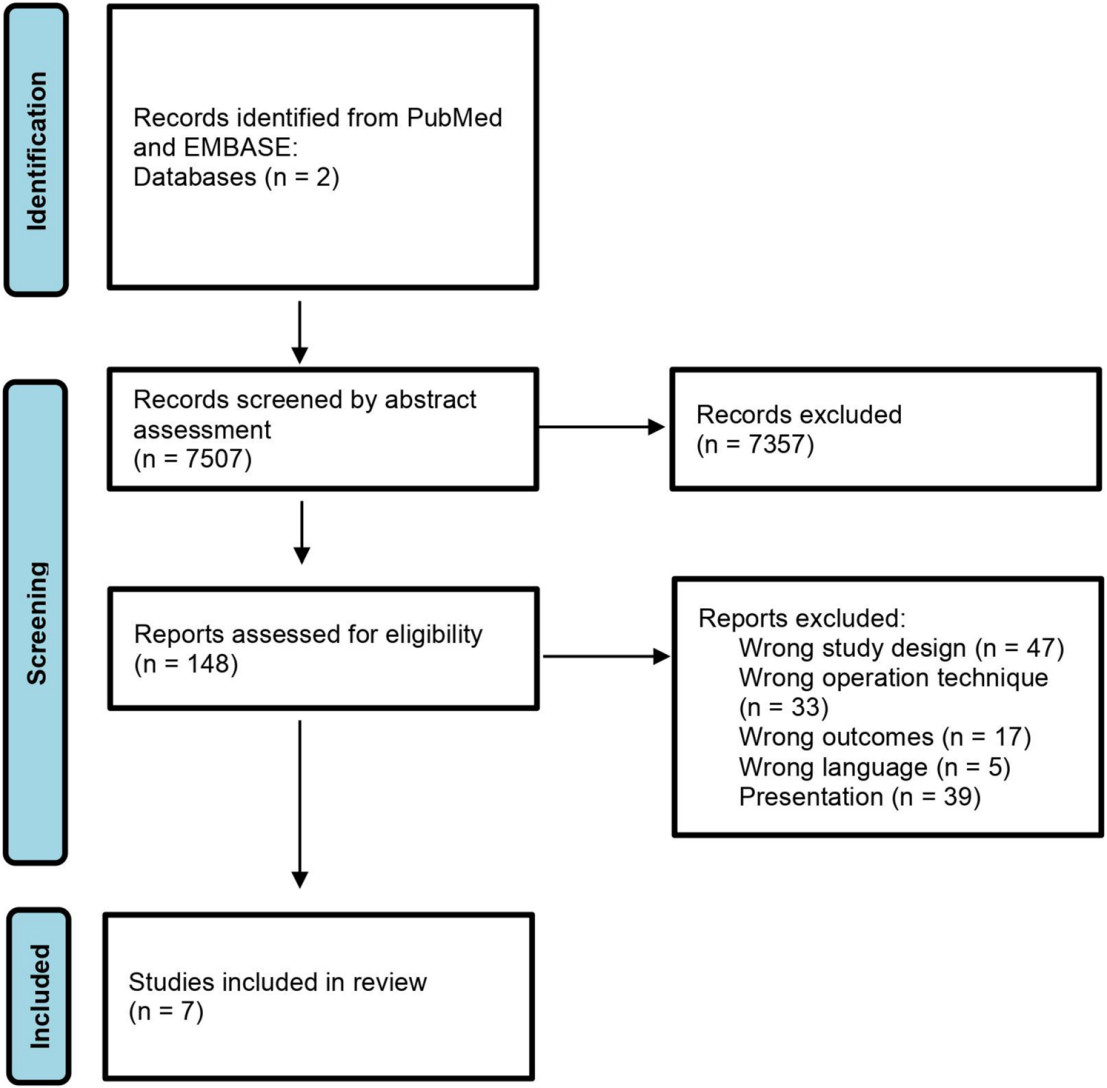
PRISMA 2020 flow diagram showing article selection

Abstracts were assessed unblinded for eligibility by the authors KAR and HL. Next, the full papers of the selected abstracts were obtained and evaluated. Consensus between the two authors was obtained concerning any disagreements regarding study inclusion. The study selection process is provided in Fig. 1.

Among the articles for which the full papers were acquired, the reference lists were examined in order to identify additional eligible studies. There was no need to contact the authors of the trials for further elaborations or clarifications. ClinicalTrials.gov was searched on 26th of February 2021 for on-going trials using the term “laparoscopic hernia repair.”

The current review including meta-analysis did not require Institutional Review Board (IRB) approval or written content.

### Risk of bias in individual studies

To evaluate the risk of bias for each eligible paper, the revised Cochrane risk of bias tool for randomized trials (RoB 2.0) [9, 10] was used. The trials were assessed for risk of bias addressing the following five domains: bias arising from the randomization process, bias due to deviations from intended interventions, bias due to missing outcome data, bias in measurement of the outcome, and bias in selection of the reported result. Assessment of the bias was classified as “low risk,” “some concerns,” or “high risk.” The risk of bias assessment was performed individually for both outcomes [hernia recurrence (Table 2) and CPIP (Table 3)], since assessment may be affected by the outcome.

The quality of the evidence was assessed using the Grading of Recommendations Assessment, Development and Evaluation (GRADE) tool for outcomes in the meta-analysis [11]. Based on the overall assessment the quality was divided into four grades (high, moderate, low or very low). The trials were either downgraded or upgraded in quality depending on whether the criteria of risk of bias, inconsistency, indirectness, imprecision, publication bias, large magnitude, dose response or effect of all plausible confounding factors were meet. Authors KAR and HCP performed the GRADE assessment.

### Meta-analysis

For the meta-analysis Review Manager (RevMan) [Computer program] (Version 5.3. Copenhagen: The Nordic Cochrane Centre, The Cochrane Collaboration, 2014) was used.

For continuous outcome (VAS or NRS), the pooled estimate was calculated using inverse variance methods and reported as mean difference with 95% confidence intervals (CI). For dichotomous outcome (recurrence), the Mantel–Haenszel method was used and odds ratio with 95% CI was reported.

To account for heterogeneity, the random effect model was used. Heterogeneity was calculated using the *χ*^2^ test with significance level set at *p* < 0.05, and was quantified using *I*^2^ with a maximum value of 30% identifying low heterogeneity [12]. A forest plot was used to display the results with a square around the estimate for the accuracy of the estimation.

## Results

A total of 7507 publications were identified. After applying the eligibility criteria, seven prospective, randomized controlled trials [13–19] were included. Laparoscopic TAPP inguinal hernia repair was performed in 1732 patients with 737 procedures performed without fixation of the mesh and 995 procedures with fixation. Follow-up period ranged from 3 to 21 months in the different trials. Baseline characteristics and follow-up period of the included trials are provided in Table 1.

**Table 1:** Baseline patient characteristics for each of the included trials

Trial	Age, years		Weight (kg/m^2^ (BMI) or kg)		Sex, *n* (male/female)		Follow-up period	
	Without fixation	With fixation	Without fixation	With fixation	Without fixation	With fixation	Without fixation	With fixation
Cambal M [14]	Mean ± SD 52.6 ± 14.9	Mean ± SD 50.3 ± 15.8			41/9	37/13	2 days: 50 out of 50 patients* 1 month: 48 out of 50 patient* 3 months: 47 out of 50 patient*	2 days: 50 out of 50 patients* 1 month: 49 out of 50 patient* 3 months: 49 out of 50 patient*
Ferrarese A [15]	Mean ± SD 53.3 ± 10.9	Mean ± SD 53 ± 11.0	Mean BMI ± SD 24.1 ± 1.6	Mean BMI ± SD 24.5 ± 1.2	30/0	30/0	Mean follow-up period: 11 months	
Li W [17]	Mean ± SD (range) 43.6 ± 14.1 (19–78)	Mean ± SD (range) 2.5 ± 12.8 (19–76)	Mean BMI ± SD (range) 24.0 ± 5.3 (20.5–36.1)	Mean BMI ± SD (range) 24.3 ± 4.1 (20.1–32.2)	50/0	50/0	Mean follow-up period ± SD 11.2 ± 2.9 months	Mean follow-up period ± SD 11.2 ± 3.6 months
Romario UF [16]	Mean (range) 61 (35–77)	Mean (range) 55 (38–75)	Median BMI (range) 24 (18–31)	Median BMI (range) 26 (19–34)	45/5	46/0	Mean follow-up period (range) 10 months (2–42)	Mean follow-up period (range) 21 months (5–27)
Smith AI [18]	Median (range) 53 (14–85)	Median (range) 54 (15–86)	Median kg (range) 78 (60–110)	Median kg (range) 76 (59–120)	247/6	239/10	Mean follow-up period (range) 17 months (3–29)	
Wang L [13]	Median (range) 47 (16–78)						Mean follow-up period (range) 15 months (6–24)	
Habeeb T [19]	Range** 20–50				260/6	511/21	Mean follow-up period 18 months	

*The trial does not report mean or median follow-up period

**The trial does not report mean or median age

### Hernia recurrence

**Fig. 2:**
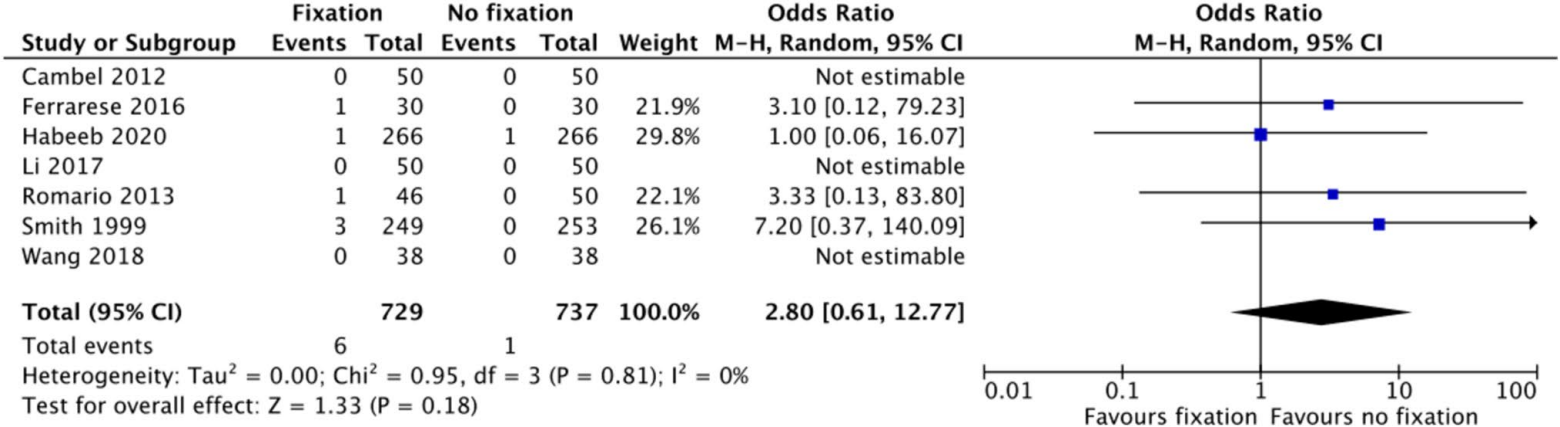
Meta-analysis of hernia recurrence

All seven trials [13–19] reported hernia recurrence and were included in meta-analyses with 1732 patients. Laparoscopic inguinal hernia repair without mesh fixation was performed on 737 patients with only two documented hernia recurrences (Table 4). Laparoscopic inguinal hernia repair with mesh fixation was performed on 995 patients. Among these, eight patients had a recurrence of their inguinal hernia. The meta-analysis for these trials found a pooled OR of 2.80 (95% CI 0.61–12.77, Fig. 2), suggesting no significant difference in hernia recurrence between the two groups. An additional meta-analysis, performed for three trials [13, 18, 19] with at least 1-year follow-up, found a pooled OR of 1.45 (95% CI 0.28–7.46, Online Appendix 2) confirming the results from the primary meta-analysis. Additionally, sensitivity analysis regarding hernia recurrence was performed including only trials with a low overall risk of bias [15, 17, 19]. A pooled OR of 1.61 (95% CI 0.20–13.30, Online Appendix 3) was found, confirming the results from the primary meta-analysis.

### Chronic postoperative inguinal pain

**Fig. 3:**
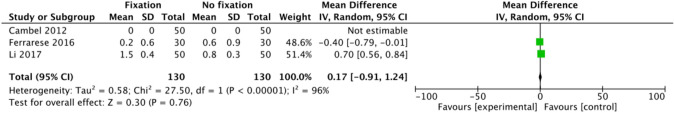
Meta-analysis of CPIP

Three of the seven trials [14, 15, 17] reported CPIP with the use of VAS or NRS and were included in meta-analyses with 260 patients. The meta-analysis of these trials generated a pooled mean difference of 0.17 (95% CI 0.90–1.24) (Fig. 3), suggesting no significant difference in CPIP between the two groups. Sensitivity analysis regarding trials with low risk of bias was not performed regarding CPIP because only one trial had a low overall risk of bias [15]. The trial showed no statistically significant differences in CPIP at 1 day, 7 days or 3 months postoperative between the groups using the VAS score, confirming the results from the primary meta-analysis.

#### Risk of bias—hernia recurrence

All seven trials were reported as randomized, even though only four of the seven trials described a random component in their sequence generation process and only three of the seven trials reported their process of concealment. This resulted in an overall assessment of some concerns regarding bias arising from the randomization process in two of the seven trials and a high risk of bias in two of the seven trials.

Bias due to missing outcome data was considered high risk in one of the seven trials due to considerable different follow-up periods in their two study groups, resulting in missing data regarding hernia recurrence in the group with the short follow-up period.

Regarding bias due to deviations from intended interventions, bias in measurement of the outcome and bias in selection of the reported result, the overall risk of bias in all three domains was low regarding hernia recurrence.

Overall, despite being RCTs, the trials were limited by considerable bias and the overall risk of bias regarding hernia recurrence was high. An overview of the risk of bias assessment using the ROB-2 tool is presented in Table 2 for hernia recurrence. Detailed justifications for the bias analysis are shown in Online Appendix 4.

**Table 2:**
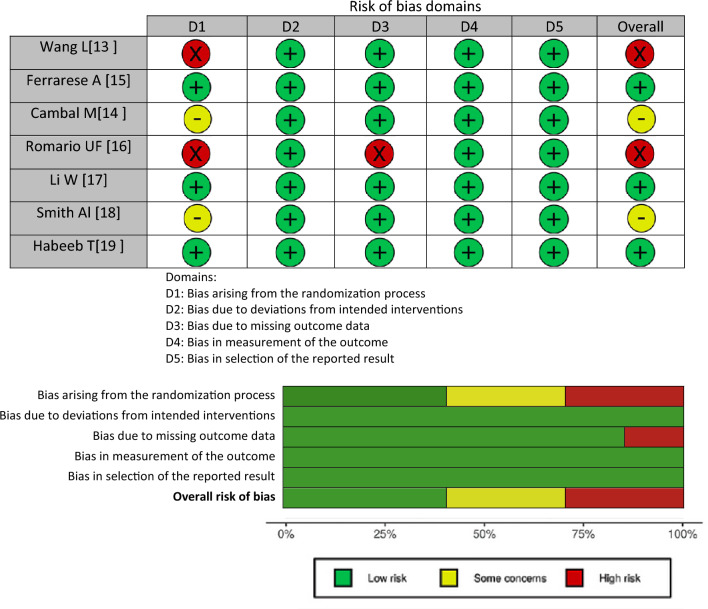
Risk of bias assessment of RCTs using the ROB-2 tool for hernia recurrence. (a) Traffic light plot of RCT bias assessment. (b) Weighted summary plot of the overall type of bias encountered in RCTs

#### Risk of bias—chronic postoperative inguinal pain

Assessment of bias arising from the randomization process was considered of some concerns in one of the two trials due to lack of information regarding concealment of allocated intervention regarding the study participant.

Regarding bias due to deviations from intended interventions and bias due to missing outcome data, the overall risk of bias for both domains was low for CPIP.

Regarding bias in measurement of the outcome, the outcome assessors for CPIP (a patient reported outcome) were the study participants. Due to lack of information regarding awareness of allocated intervention, the risk of bias was considered high in two of the three trials since knowledge of assigned intervention may influence the outcome.

Risk of bias in selection of the reported result was high in one of the three trials due to lack of fully reporting all results from the trial regarding this outcome.

Overall, the trials were limited by substantial bias and the overall risk of bias regarding CPIP was high. An overview of the risk of bias assessment using the ROB-2 tool is presented in Table 3 for CPIP. Detailed justifications for the bias analysis are shown in Online Appendix 5 (Table 4).

**Table 3:**
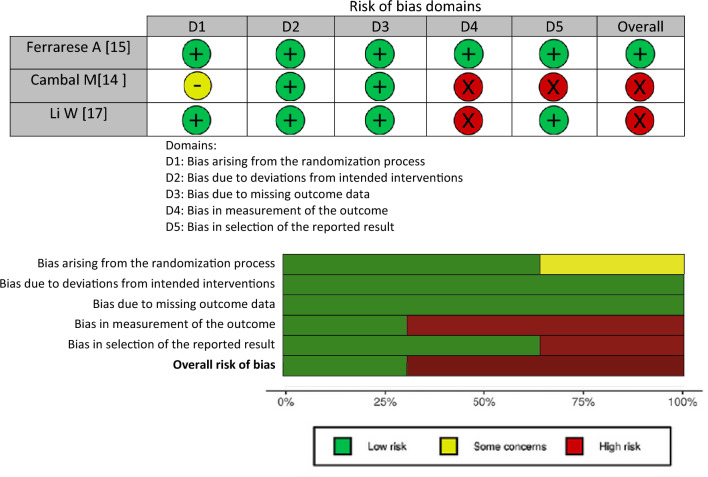
Risk of bias assessment of RCTs using the ROB-2 tool for chronic postoperative inguinal pain. (a) Traffic light plot of RCT bias assessment. (b) Weighted summary plot of the overall type of bias encountered in RCTs

**Table 4:** Variables used for meta-analysis

Trial	Patients (*n*)		Mean VAS or NRS (1–10 ± SD)		Recurrence (*n* = %)	
	Without fixation	With fixation	Without fixation	With fixation	Without fixation	With fixation
Cambal M [14]	50	50	3 months 0 ± 0	3 months 0 ± 0	0/50 = 0%	0/50 = 0%
Ferrarese A [15]	30	30	1 day 2.4 ± 0.8 7 days 1.0 ± 1.0 3 months 0.6 ± 0.9	1 day 2.5 ± 0.8 7 days 1.1 ± 0.9 3 months 0.2 ± 0.6	0/30 = 0%	1/30 = 0.3%
Li W [17]	50	50	2 days 3.6 ± 0.5 (range 2–8) 3 months 0.8 ± 0.3 (range 0–4) 6 months 0.8 ± 0.3 (range 0–4)	2 days 4.5 ± 0.9 (range 2–8) 3 months 1.5 ± 0.4 (range 0–5) 6 months 1.0 ± 0.1 (range 0–3)	0/50 = 0%	0/50 = 0%
Romario UF [16]	50	46	*		0/50 = 0%	1/46 = 2.2%
Smith AI [18]	253	249	*		0/253 = 0%	3/249 = 1.2%
Wang L [13]	38	38	*		0/38 = 0%	0/38 = 0%
Habeeb T [19]	266	532	*		2/266 = 0.8%	3/532 = 0.6%

*Did not use or report results about mean VAS scores to describe CPIP

#### Quality of evidence on outcome level—hernia recurrence (Table 5)

Due to design of studies (RCTs), the quality of evidence for hernia recurrence was initially high. However, due to serious limitations regarding risk of bias the outcome was graded down to moderate. The serious limitations in the Risk of bias category was assessed due to the fact that two of seven studies with high risk of bias were included in the meta-analyses with a weight of 26.1% [18] and 22.1% [16], respectively. Furthermore, two of seven studies had some concern regarding risk of bias (Table 2). Furthermore, we found evidence of serious imprecision due to wide confidence interval. In addition, the estimated absolute effect was very small (2 more per 1000) leading to a number needed to treat of 500 patients. Thus, supporting further downgrading to low. We found no indication of inconsistency (heterogeneity measured by *I*^2^ = 0%), indirectness (population, intervention, comparator, and outcomes were comparable between studies) or publication bias (symmetric funnel plot).

**Table 5:** Quality of evidence on outcome level

Certainty assessment							No of patients		Effect		Certainty	Importance
No of studies	Study design	Risk of bias	Inconsistency	Indirectness	Imprecision	Other considerations	mesh fixation	Non-fixation of mesh	Relative (95% CI)	Absolute (95% CI)		
Chronic postoperative inguinal pain [assessed with: Visual Analogue Scale (VAS)]												
3	Randomized trials	Very serious	Serious^a^	Not serious	Serious^b^	None	130	130	–	MD 0.17 higher (0.91 lower to 1.24 higher)	⨁◯◯◯ Very low	Critical ^f^
Recurrence (assessed with: Clinical assessment)												
7	Randomized trials	Serious^c^	Not serious	Not serious	Serious^d^	None	6/729 (0.8%)	1/737 (0.1%)	OR 2.80 (0.61 to 12.77)	2 more per 1.000 (from 1 fewer to 16 more)	⨁⨁◯◯ Low	Important ^e^

*CI* confidence interval, *MD* mean difference, *OR* odds ratio

^a^High heterogeneity (96%)

^b^Wide confidence intervals, small effect

^c^High risk of bias in 2/7 studies, some concern in 2/7 studies

^d^Wide confidence intervals, small effect

^e^Chronic pain may be devastating for the patients' quality of life, more so than a possible recurrence

^f^Recurrence is important. However, depending on symptoms less of a problem compared with chronic pain

#### Quality of evidence on outcome level—chronic postoperative inguinal pain (Table 5)

The high quality of evidence due to study design was downgraded to low due to very serious risk of bias in that six of seven studies for this outcome had high risk of bias. Among the studies included in the meta-analysis, two of three studies had high risk of bias (Table 3). The quality was further downgraded to very low due to serious inconsistency. This was due to a very high heterogeneity between the studies (*I*^2^ = 96%). The high heterogeneity arose from the fact that two studies from opposite directions of the effect. Li et al. [17] found lower pain level with no fixation, whereas Ferrarese et al. [15] found the opposite effect. Based on a previous study, the minimal clinically relevant difference in VAS score was estimated to be 10 mm [20]. Considering our confidence intervals (95% CI  0.90–1.24) for CPIP, the sample may be too small to rule out a possible clinically relevant difference. Furthermore, the estimated absolute effect of the intervention was very small (0.17 VAS). Based in this, we have assessed serious imprecision for this outcome. Lastly, there was no indication of indirectness (population, intervention, comparator, and outcomes were comparable between studies) or publication bias (symmetric funnel plot).

## Discussion

Based on the available evidence, we could not detect a difference in hernia recurrence in patients undergoing laparoscopic inguinal hernia repair using the TAPP approach based on whether the applied mesh was fixated or not. Similarly, we did not detect any difference in the risk of developing CPIP. However, there was high risk of bias in 2/7 and 2/3 trials for the two outcomes hernia recurrence and CPIP, respectively. The quality of evidence for the outcomes, hernia recurrence and CPIP, were low and very low according to the GRADE criteria (Table 5).

Concerning the hernia recurrence rate, we found a recurrence rate of 0.3% after laparoscopic inguinal hernia repair without mesh fixation in all included trials. This is unusually low, as many previous trials have shown recurrence rates after TAPP in up to 15% of cases [21]. However, this could be due to the method of evaluating hernia recurrence or especially the follow-up period. Only four trials [13, 16, 18, 19] had a follow-up period of more than 12 months, whereas the remaining trials had a follow-up period between 3 and 11 months. One trial even had different mean follow-up periods between their two study groups (10 months mean follow-up in the group without fixation and 21 months in the fixated group) [16]. In regard to CPIP, the different mean follow-up periods would not have an effect on the outcome, because CPIP was assessed at a 6-month follow-up visit for both groups, where a comparable number of patients from the two groups were included. In addition, the trial was not included in our meta-analysis for CPIP because it used McGill Pain Questionnaire to assess CPIP. In contrast, the different mean follow-up periods may influence the result regarding hernia recurrence. There is a risk of underreporting hernia recurrence in the group with the short mean follow-up period because the data for the “remaining” follow-up period was missing. This was reflected in our bias assessment of the outcome.

Based on the additional meta-analysis of the trials with at least 1-year follow-up, there were still no differences in recurrence between mesh fixation and non-fixation of the mesh. Furthermore, all studies, except two [18, 19], had small sample sizes, which is critical when investigating rare events. Furthermore, sensitivity analysis of studies with low risk of bias did not significantly affect the estimate.

Regarding CPIP, there was an overall lack of consistency regarding how CPIP was defined and reported. Four trials were excluded from the analysis because they either did not the VAS or NRS or results regarding VAS or NRS were not reported about CPIP. There is no consensus on the precise definition of CPIP. However, there is some agreement that CPIP may be defined as on-going pain over a period of at least 3 months [7, 22]. Most included studies had a follow-up period of at least 3 months. The pathophysiology behind CPIP is not fully elucidated. Preoperative pain has been found to be a strong predictor of CPIP [23], whereby the surgical procedure itself may not fully explain the occurrence and rate of CPIP. Furthermore, CPIP may develop regardless of fixation method, which points to the dissection itself and/or the mesh to be the cause of pain and not the fixation.

In this systematic review and meta-analysis, we aimed to include the highest level of evidence available. We feared that including non-randomized trials would introduce substantial bias in the review with risk of distorting the results. With 7 relatively homogenous RCTs including 1732 patient, we felt the included trials provided sufficient data for a robust review. Despite low quality of the evidence, there was no important disagreement between the studies. As seen in Fig. 1, 47 studies were excluded due to wrong study design. In addition, studies on non-inguinal groin hernias were excluded to avoid heterogeneity in the population that may limit the clinical applicability of the study. Thus, the effect of fixation of the mesh may not necessarily be comparable between types of groin hernias. Another aspect to consider is that only one trial specifically reported a cut-off regarding size of the hernia [17], whereas the other trials did not have a cut-off in their inclusion/exclusion criteria. Moreover, we recognize that time-sensitive outcomes are best assessed using time-to-event data. However, this was not feasible due to the way data were reported in the studies. Regarding inclusion criteria in this review, recurrence was defined based on clinical exam and chronic pain based on visual analog score, because both methods were the commonly used methods for reporting either recurrence or chronic pain. The visual analog score was also the most comparable score across the trials. Other trials used different methods to report pain, which weren’t comparable, for example “Return to work” in days or “number of patients using analgesics in the postoperative period.” Only one study used the McGill Pain Questionnaire[16, 24].

Gender ratio was uneven in two of the seven trials where only male participants were included [15, 17] and one trial did not specify the gender distribution [13]. These populations do not reflect the gender distribution in the background population with inguinal hernias. In addition, females, who have a higher risk of CPIP, are underrepresented in the included studies [25–28].

In this review, neither types of mesh nor method of mesh fixation were analyzed. Only one trial specifically reported this by comparing three groups in relation to methods of mesh fixation (i.e., no fixation, by tacker (non-absorbable and absorbable), and by histoacryl) [19]. Interestingly, they found a significant difference regarding chronic pain, but not regarding recurrence, between the three groups.

Future clinical trials are needed with emphasis on reducing the risk of bias through a proper design, sample size, follow-up period, description of methods, and determination of outcomes. Thus, properly designed RCTs with large sample sizes and long follow-up periods (minimum of 12 mounts) would be preferable.

## Conclusion

The current evidence is very uncertain and mesh fixation may have little to no effect regarding hernia recurrence and chronic postoperative inguinal pain in patients operated with TAPP inguinal hernia repair.

### Supplementary Information

 Below is the link to the electronic supplementary material.

Electronic supplementary material 1 (DOCX 12 kb)

Appendix 2: Meta-analysis of recurrence at least 1-year follow-up (JPEG 163 kb)

Appendix 3: Meta-analysis of hernia recurrence for trials with overall low risk of bias (JPG 221 kb)

Electronic supplementary material 4 (DOCX 19 kb)

Electronic supplementary material 5 (DOCX 16 kb)
