# Supplementary material for: No evidence for fixation of mesh in laparoscopic transabdominal preperitoneal (TAPP) inguinal hernia repair: a systematic review and meta-analysis of randomized controlled trials
Source: Surg Endosc. 2023 Sep 6;37(11):8291–300. doi: 10.1007/s00464-023-10237-0 (PMC10615908; doi:10.1007/s00464-023-10237-0)
Supplement: Supplementary file 1 — Electronic supplementary material 1 (DOCX 12 kb) [file 464_2023_10237_MOESM1_ESM.docx]

Appendix 1: Search strings used in PubMed and Embase

The search string used in PubMed:

*(((((((((((((((((laparoscop*[Title/Abstract]) OR transabdominal preperitoneal patch plasty[Title/Abstract]) OR tapp[Title/Abstract]) AND inguinal[Title/Abstract]) OR femoral[Title/Abstract]) OR groin[Title/Abstract]) AND hernia[Title/Abstract]) OR hernia[MeSH Terms]) AND repair[Title/Abstract]) OR herniotomy[Title/Abstract]) AND recurrence[Title/Abstract]) OR complication*[Title/Abstract]) OR pain[Title/Abstract]) AND self-adhe*[Title/Abstract]) OR adhe*[Title/Abstract]) OR cousin[Title/Abstract]) OR fixation[Title/Abstract]) AND mesh.*

The search string used in EMBASE:

((((((((((laparoscop* or transabdominal preperitoneal patch plasty or tapp) and inguinal) or femoral or groin) and hernia and repair) or herniotomy) and recurrence) or complication* or pain) and self-adhe*) or adhe* or cousin or fixation) and mesh).af.
