# Supplementary figures and images for: No evidence for fixation of mesh in laparoscopic transabdominal preperitoneal (TAPP) inguinal hernia repair: a systematic review and meta-analysis of randomized controlled trials
Source: Surg Endosc. 2023 Sep 6;37(11):8291–300. doi: 10.1007/s00464-023-10237-0 (PMC10615908; doi:10.1007/s00464-023-10237-0)

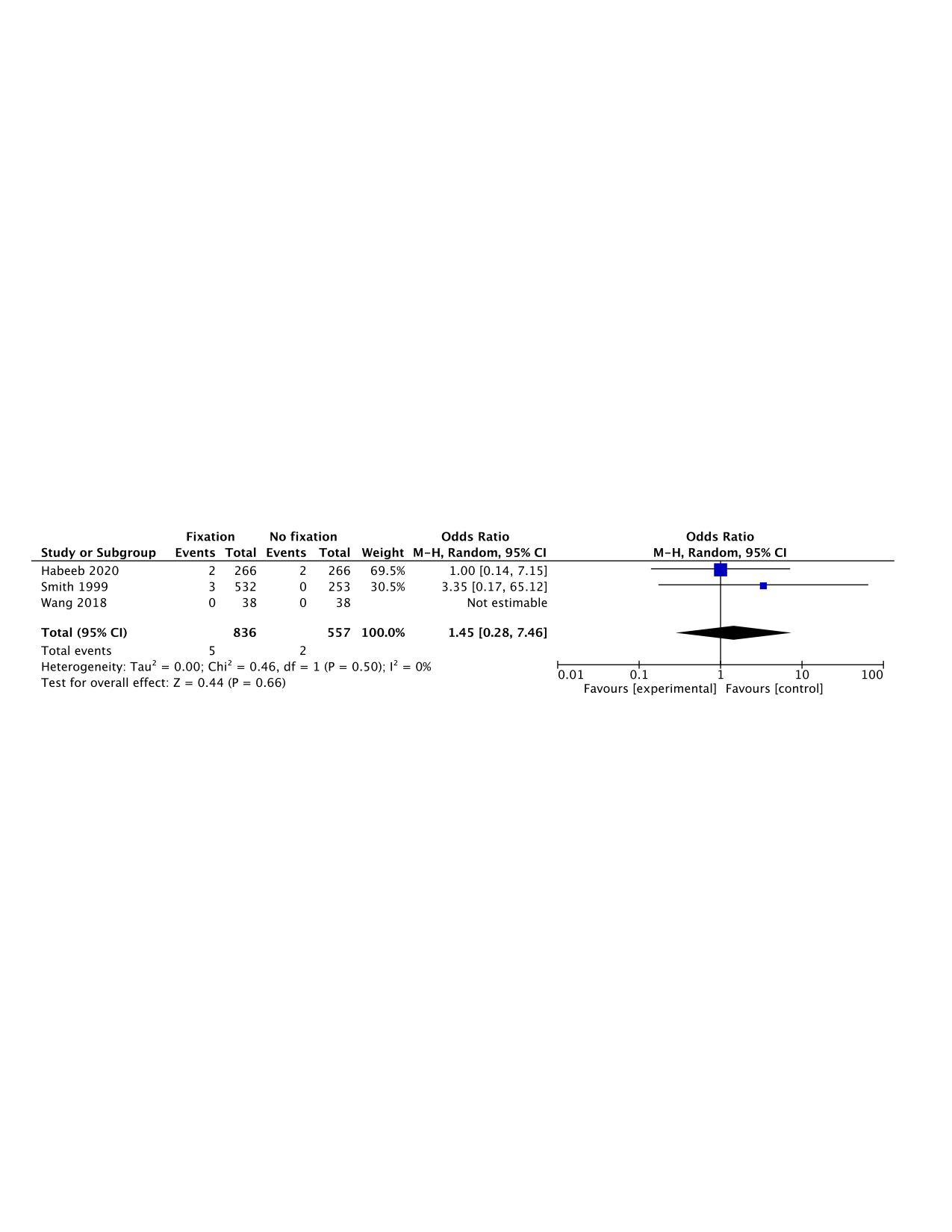

Supplement: Supplementary file 2 — Appendix 2: Meta-analysis of recurrence at least 1-year follow-up (JPEG 163 kb) [file 464_2023_10237_MOESM2_ESM.jpeg]

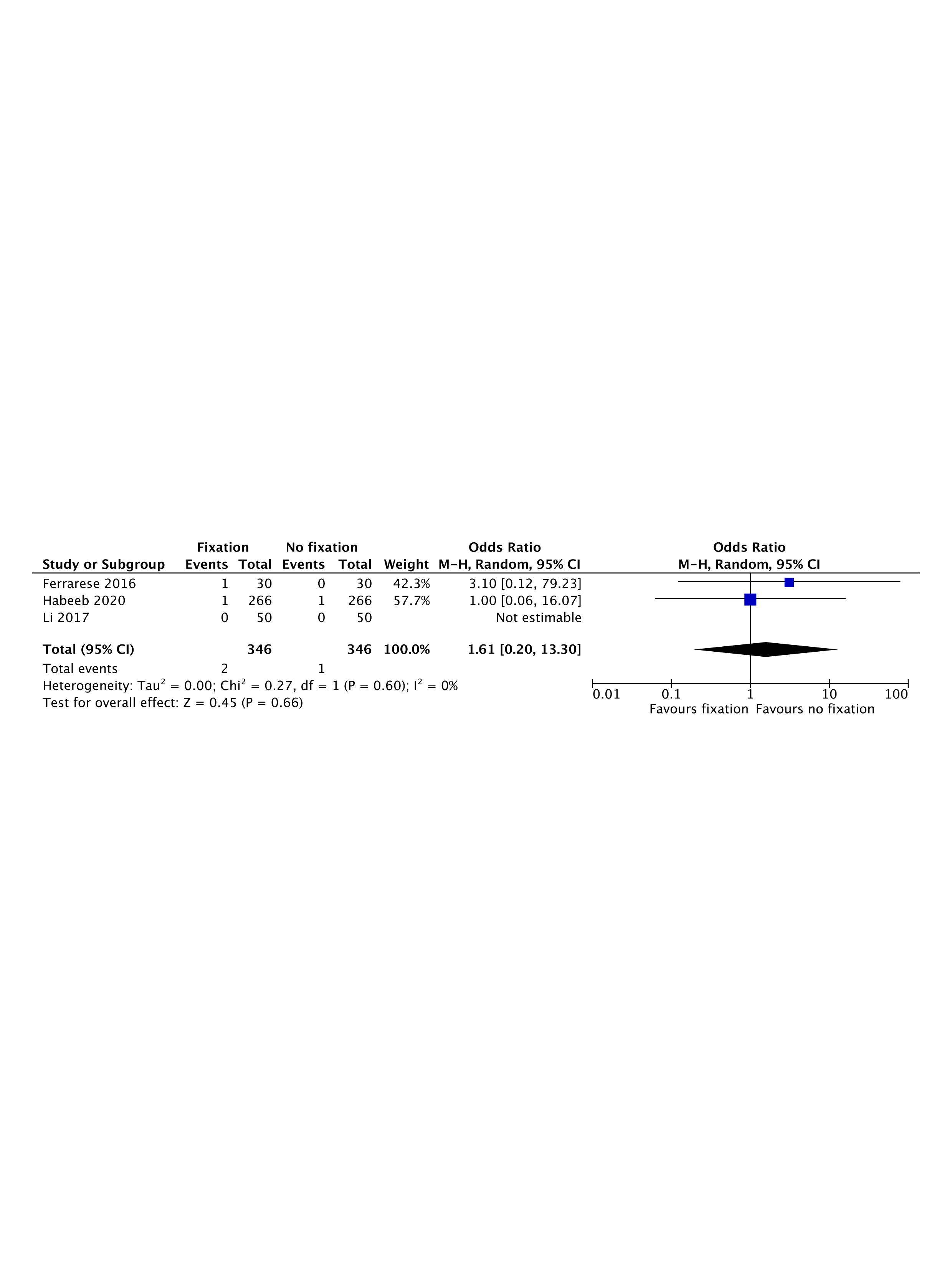

Supplement: Supplementary file 3 — Appendix 3: Meta-analysis of hernia recurrence for trials with overall low risk of bias (JPG 221 kb) [file 464_2023_10237_MOESM3_ESM.jpg]
