# Supplementary material for: No evidence for fixation of mesh in laparoscopic transabdominal preperitoneal (TAPP) inguinal hernia repair: a systematic review and meta-analysis of randomized controlled trials
Source: Surg Endosc. 2023 Sep 6;37(11):8291–300. doi: 10.1007/s00464-023-10237-0 (PMC10615908; doi:10.1007/s00464-023-10237-0)
Supplement: Supplementary file 5 — Electronic supplementary material 5 (DOCX 16 kb) [file 464_2023_10237_MOESM5_ESM.docx]

Appendix 5: Detailed justifications for the bias analysis regarding chronic postoperative inguinal

Risk of bias judgements for bias arising from the randomization process (D1)

|  | **Signalling question** | | | **Domain level judgement** |
| --- | --- | --- | --- | --- |
|  | **1.1** | **1.2** | **1.3** | **Risk of bias** |
| **Ferrarese A (15)** | Y | Y | N | Low risk |
| **Cambal M (14)** | Y | NI | N | Some concerns |
| **Li W (17)** | Y | Y | N | Low risk |

Y/PY = “Yes” or “Probably yes”; N/PN = “No” or “Probably no”; NI = “No information”;

NA = Not applicable

Risk of bias due to deviations from intended interventions (D2)

|  | **Signalling question** | | | | | | **Domain level judgement** |
| --- | --- | --- | --- | --- | --- | --- | --- |
|  | **2.1**  **Patient aware?** | **2.2 Personnel aware?** | **2.3**  **Any deviations?** | **2.4**  **Unbalanced deviations?** | **2.5**  **Wrong group?** | **2.6**  **Effect outcome** | **Risk of bias** |
| **Ferrarese A (15)** | N | N | NA | NA | N | NA | Low risk |
| **Cambal M (14)** | NI | NI | PN | NA | N | NA | Low risk |
| **Li W (17)** | NI | N | PN | NA | N | NA | Low risk |

Y/PY = “Yes” or “Probably yes”; N/PN = “No” or “Probably no”;

NI = “No information”; NA = Not applicable

Risk of bias due to missing outcome data (D3)

|  | **Signalling question** | | | **Domain level judgement** |
| --- | --- | --- | --- | --- |
|  | **3.1** | **3.2** | **3.3** | **Risk of bias** |
| **Ferrarese A (15)** | Y | NA | NA | Low risk |
| **Cambal M (14)** | Y | NA | NA | Low risk |
| **Li W (17)** | Y | NA | NA | Low risk |

Y/PY = “Yes” or “Probably yes”; N/PN = “No” or “Probably no”;

NI = “No information”; NA = Not applicable

Risk of bias in measurement of the outcome (D4)

|  | **Signalling question** | | **Domain level judgement** |
| --- | --- | --- | --- |
|  | **4.1** | **4.2** | **Risk of bias** |
| **Ferrarese A (15)** | N | NA | Low risk |
| **Cambal M (14)** | NI | PY | High risk |
| **Li W (17)** | NI | PY | High risk |

Y/PY = “Yes” or “Probably yes”; N/PN = “No” or “Probably no”;

NI = “No information”; NA = Not applicable

Risk of bias in selection of the reported result (D5)

|  | **Signalling question** | | **Domain level judgement** |
| --- | --- | --- | --- |
|  | **5.1** | **5.2** | **Risk of bias** |
| **Ferrarese A (15)** | N | PN | Low risk |
| **Cambal M (14)** | Y | NI | High risk |
| **Li W (17)** | N | PN | Low risk |

Y/PY = “Yes” or “Probably yes”; N/PN = “No” or “Probably no”;

NI = “No information”; NA = Not applicable
